# Prevalence and predictors of bacterial vaginosis in HIV-infected women in Maharashtra, India

**DOI:** 10.1177/0956462419878333

**Published:** 2020-03-31

**Authors:** S Joshi, A Mane, R Muwonge, U Divate, V Padbidri, V Kulkarni, R Gangakhedkar, R Sankaranarayanan

**Affiliations:** 1 Hirabai Cowasji Jehangir Medical Research Institute and Prayas, Pune, India; 2 National AIDS Research Institute, Pune, India; 3 Screening Group, Early Detection & Prevention Section, International Agency for Research on Cancer, Lyon, France; 4 Hirabai Cowasji Jehangir Medical Research Institute, Pune, India; 5 Prayas, Amrita Clinic, Pune, India; 6 National AIDS Research Institute, Pune, India; 7 Research Triangle Institute, International-India, New Delhi, India

**Keywords:** Bacterial vaginosis, HIV, human papillomavirus

## Abstract

We evaluated the prevalence and determinants of bacterial vaginosis (BV) in HIV-infected women from Maharashtra, India. Among 912 HIV-infected women enrolled, BV was diagnosed in 191 (20.9%) and intermediate BV was diagnosed in 258 (28.3%) women. Women with more than two pregnancies had 1.6 times increased risk of BV (95% CI 1.0, 2.5, p-value 0.038), women who were menopausal had 6.2 times increased risk of BV (95% CI 2.4, 15.6, p-value <0.001) and women who were human papillomavirus (HPV) positive had 2.3 times increased risk of BV (95% CI 1.4, 3.9, p-value 0.001). Although we observed significantly increased risk of BV among women diagnosed with cervical intraepithelial neoplasia or worse disease in the univariate analysis (odds ratio 3.5, 95% CI 1.5, 8.1, p-value 0.004), it did not reach statistical significance in the multivariate analysis. Women who had the first sexual intercourse after the age of 18 had significantly lower risk of BV. To conclude, we observed high prevalence of BV in HIV-infected women and increased risk of BV in HPV positive, HIV-infected women.

## Introduction

Among the women of reproductive age, bacterial vaginosis (BV) is the most common cause of vulvovaginitis.^1^ The aetiopathology of BV is still being debated and the epidemiologic data support sexual transmission; however, disagreement exists.^2^ BV may be considered a sexually enhanced disease, with frequency of intercourse being a critical factor.^3^ Associations between BV and factors such as ethnicity, intra-vaginal douching, new sexual partners, multiple sex partners and unprotected vaginal intercourse have been reported.^4,5^ Globally, the prevalence of BV varies considerably between countries, regions within countries and ethnic groups, and it can be as high as 60% in certain regions.^6^ This variation is because of the differences in the biological, behavioural, medical, social and economic factors.^2^

BV is characterized by an imbalance in the normal vaginal flora. The normal vaginal flora consists of lactic acid- and hydrogen peroxide (H_2_O_2_)-producing lactobacilli. BV is associated with reduction in the normal vaginal flora and concurrent increase in vaginal anaerobic flora. Clinically, BV is characterized by the presence of three of the four criteria which include discharge (thin, homogenous, uniformly adherent, white discharge), vaginal pH > 4.5, fishy odour on addition of 10% potassium hydroxide (KOH) and 20% clue cells (epithelial cell margins obscured by bacteria) on microscopic examination of vaginal smear.^7^ However, about 50% of the women with BV do not have any symptoms,^8^ hence its control is difficult and it remains untreated. Therefore, a standardized scoring system for the interpretation of Gram-stained vaginal smears called as Nugent’s scoring has been introduced.^9^ Nugent’s scoring is the gold standard for the laboratory diagnosis of BV.

BV has been associated with an increased risk of human immunodeficiency virus (HIV) acquisition^10^ and transmission.^11^ But the studies that concluded increased risk of HIV transmission to the HIV-uninfected partner were done when women living with HIV did not receive highly active antiretroviral therapy (HAART) which reduces the HIV transmission significantly.^12^ A recent study from Denmark has shown that in women who are on HAART, presence of BV, human papillomavirus (HPV) and herpes viridae does not predict vaginal HIV RNA shedding implying that HIV shedding is not increased by BV in women on HAART.^12^ But women with BV are at an increased risk of acquiring various other sexually transmitted infections (STIs) such as herpes simplex virus type 2,^13^ *Trichomonas vaginalis*, *Neisseria gonorrhoeae*^14–16^ and *Chlamydia trachomatis*.^16^ This increased risk is possibly because of the inflammation of the genital mucosa.^17^ BV also increases the risk of adverse obstetric and gynaecological outcomes such as preterm delivery, complications of gynaecological surgeries and pelvic inflammatory disease.^18,19^

A high prevalence of BV between 35 and 49% has been reported from a large study conducted among more than 37,000 women in sub-Saharan Africa.^20^ A meta-analysis of studies for the risk factors of BV has reported that BV is significantly associated with sexual contact with a new partner or multiple sexual partners and that decreasing the number of unprotected sexual encounters may reduce incident and recurrent infection.^4^

Very few studies in India have reported BV prevalence among women and it was 8.6 and 20.5% among pregnant women attending two different antenatal clinics in North India, respectively.^21,22^ The prevalence of BV ranged from 17 to 19% among women from the general population^23,24^ and among female sex workers, it was 45%.^25^ To our knowledge, only one study in India has reported the prevalence of BV in HIV-infected women and it was 47.7%.^26^ Having highlighted the negative effects of BV on the reproductive health and the evidence suggesting BV and HIV/STI control plus a dearth of data on this aspect in India, we are reporting the prevalence and risk factors of BV in HIV-infected women from a cross-sectional study conducted in Maharashtra, India.

## Material and methods

The study was reviewed and approved by the ethical review committees of Hirabai Cowasji Jehangir Medical Research Institute (HCJMRI) and Prayas Health Group, Pune, India and that of the International Agency for Research on Cancer of the WHO, Lyon, France. Screening was initiated on 9 September 2010 and completed on 3 November 2011. Our study procedures have been described earlier.^27^ The study was conducted at a designated study clinic in Pune, India where consecutive, serologically confirmed HIV-infected women were enrolled in the study.

We contacted physicians treating HIV-infected women as well as various non-governmental organizations (NGOs) working for HIV-infected individuals in high HIV prevalent districts in Maharashtra state, and they were informed about the need for screening HIV-infected women for cervical cancer and the study procedures. Group meetings of HIV-infected women were arranged by the NGOs; a study specific social worker attended these meetings and educated women with the help of a user friendly, pictorial flip chart. Women willing to get enrolled were referred to the study clinic in groups by the NGOs and their travel expenses were reimbursed.

HIV-infected women in the age group of 21–60, having an intact uterus, who were not pregnant and had not received any prior treatment for cervical intraepithelial neoplasia (CIN) or cervical cancer, were eligible for the study. They were explained the study objectives and procedures, and their written informed consent was obtained. They were interviewed for socio-demographic, sexual, reproductive, medical and HIV infection-related characteristics using a structured questionnaire by a female social worker.

Cervical cell samples were collected following the manufacturer’s instructions (Qiagen) using a cervical brush provided by the manufacturer after exposing the cervix with a cusco vaginal speculum and the brush was placed in a specimen transport medium (STM) for HPV DNA detection by the Hybrid Capture 2 (HC2) test. A vaginal sample was collected from the lateral vaginal wall and posterior fornix with a sterile cotton swab for smear preparation and rolled on a glass slide for Nugent’s scoring.

Women presenting with any reproductive tract infection (RTI) or STI were offered treatment following the WHO guidelines for STIs.^28^ Procedures for cervical cancer screening, colposcopy, biopsy, treatment for CIN and histopathology reporting of cervical biopsy specimens have been described in our previous publications of this study.^27^

The air dried slides for Nugent’s scoring were transported to the laboratory at room temperature where they were stained by Gram stain, and reporting was done following Nugent’s criteria by a microbiologist who was blinded to the study data. Each Gram-stained smear was evaluated for the following morphotypes under oil immersion (1000× magnification): large Gram-positive rods (Lactobacillus morphotypes), small Gram-negative to Gram-variable rods (*Bacteroides* spp. and *Gardnerella vaginalis* morphotypes) and curved Gram variable rods (*Mobiluncus* spp. morphotypes). Each morphotype was quantitated from 1 to 4+ with regard to the number of morphotypes per oil immersion field (0, no morphotypes; 1+, less than 1 morphotype; 2+, 1–4 morphotypes; 3+, 5–30 morphotypes; 4+, 30 or more morphotypes) by a microbiologist. The scoring criteria summed the weighted quantitation (0, 1 to 4+) of the three morphotypes to yield a score of 0–10 for each person. The criterion for BV was a score of 7 or higher; a score of 4–6 was considered intermediate, and a score of 0–3 was considered normal.

The Gram stain reagents were checked weekly and whenever a new lot of stain was put into use. The reagents were evaluated by staining the recommended bacterial strains: *Staphylococcus aureus* ATCC 25923 and *Escherichia coli* ATTC 25922. Gram stain quality control was performed with every run of samples with the ATCC controls. The Gram stain slides were reported by an independent microbiologist who was trained using the training set of slides provided by the Microbicide Trials Network and was blinded to any other data including HPV test report or CIN status. In case of any doubt, the slides were seen by two other expert microbiologists for a consensus in reporting.

The STM containing cervical cells was tested by the HC2 assay for 13 high-risk HPV types (16, 18, 31, 33, 35, 39, 45, 51, 52, 56, 58, 59 and 68) as per the manufacturer’s instructions (Digene Corporation, USA) at the Nargis Dutt Memorial Cancer Hospital, Barshi. A positive result was recorded for specimens with a ratio of relative light unit to a positive control of 1 or more, corresponding to 5000 or more viral copies.

Data were entered using Access 2000 software and statistical analysis was carried out using STATA software, version 14.2 (StataCorp, College Station, Texas, USA). Two different endpoints were assessed that included intermediate BV and BV. Odds ratios (ORs) together with their 95% confidence intervals (CIs) obtained from multinomial logistic regression models were used to assess the effect of socio-demographic, sexual, reproductive, medical and HIV infection-related characteristics on intermediate BV and BV infection. Factors that had p-value <0.2 in the univariate analysis plus age, ART status and baseline CD4 cell count, a priori selected, were included in the multivariate regression model to determine the factors that independently affect intermediate BV and BV. In these regression analyses, participants with neither intermediate BV nor BV were used as the base outcome indicating that for the assessment of the participants’ factors affecting a particular endpoint (intermediate BV or BV), only data for participants with none of the endpoints and those with that particular endpoint were used.

## Results

The characteristics of the 912 women enrolled (mean age 34.6, SD: 6.3; range: 21–62) and the distribution of intermediate BV and BV are given in Table 1. Majority of the enrolled women (688/912, 75.4%) were between 30 and 44 years of age, 157/912 (17.2%) were less than 30 years old and 67/912 (7.3%) were above the age of 45. There were 332/912 (36.4%), 184/912 (20.2%) and 396/912 (43.4%) women from rural, semi-urban and urban areas, respectively.

**Table 1.** Characteristics of women and the distribution of intermediate BV and BV.

Patient characteristics	Women assessed	Women with intermediate BV			Women with BV		
	n	n	Percentage (95% CI)		n	Percentage (95% CI)	
Women assessed	912	258	28.3	(25.4–31.3)	191	20.9	(18.3–23.7)
Geographic location in Maharashtra							
Western Maharashtra/Desh	703	191	27.2	(23.9–30.6)	155	22.0	(19.0–25.3)
Marathwada	55	17	30.9	(19.1–44.8)	4	7.3	(2.0–17.6)
Khandesh/North Maharashtra	119	38	31.9	(23.7–41.1)	25	21.0	(14.1–29.4)
Konkan	35	12	34.3	(19.1–52.2)	7	20.0	(8.4–36.9)
Setting							
Rural	332	108	32.5	(27.5–37.9)	76	22.9	(18.5–27.8)
Semi-urban	184	51	27.7	(21.4–34.8)	36	19.6	(14.1–26.0)
Urban	396	99	25.0	(20.8–29.6)	79	19.9	(16.1–24.2)
Age (years)							
<30	157	48	30.6	(23.5–38.4)	36	22.9	(16.6–30.3)
30–44	688	188	27.3	(24.0–30.8)	140	20.3	(17.4–23.6)
45+	67	22	32.8	(21.8–45.4)	15	22.4	(13.1–34.2)
Education							
Some education	111	32	28.8	(20.6–38.2)	28	25.2	(17.5–34.4)
Illiterate	801	226	28.2	(25.1–31.5)	163	20.3	(17.6–23.3)
Marital status							
Married	457	132	28.9	(24.8–33.3)	84	18.4	(14.9–22.2)
Unmarried/widowed/separated	454	126	27.8	(23.7–32.1)	107	23.6	(19.7–27.7)
Age at first sexual intercourse							
<18	336	102	30.4	(25.5–35.6)	94	28.0	(23.2–33.1)
18+	570	156	27.4	(23.7–31.2)	96	16.8	(13.9–20.2)
Total no. of lifetime sexual partners							
1	851	241	28.3	(25.3–31.5)	178	20.9	(18.2–23.8)
2+	57	17	29.8	(18.4–43.4)	12	21.1	(11.4–33.9)
Use of tobacco							
No	771	214	27.8	(24.6–31.1)	154	20.0	(17.2–23.0)
Yes	141	44	31.2	(23.7–39.5)	37	26.2	(19.2–34.3)
Total number of pregnancies							
0–1	313	80	25.6	(20.8–30.8)	50	16.0	(12.1–20.5)
2+	599	178	29.7	(26.1–33.6)	141	23.5	(20.2–27.1)
Miscarriages							
No	501	148	29.5	(25.6–33.7)	101	20.2	(16.7–23.9)
Yes	407	108	26.5	(22.3–31.1)	89	21.9	(17.9–26.2)
Date of last menstruation							
<12 months ago	847	241	28.5	(25.4–31.6)	164	19.4	(16.8–22.2)
>12 months ago	61	16	26.2	(15.8–39.1)	24	39.3	(27.1–52.7)
Time since diagnosis of HIV infection (in years)							
1–2	183	60	32.8	(26.0–40.1)	42	23.0	(17.1–29.7)
3–4	203	61	30.0	(23.8–36.9)	48	23.6	(18.0–30.1)
5–10	404	98	24.3	(20.2–28.7)	80	19.8	(16.0–24.0)
11–16	116	38	32.8	(24.3–42.1)	19	16.4	(10.2–24.4)
Baseline absolute CD4 cell count (cells/mm^3^)							
500+	461	111	24.1	(20.2–28.2)	98	21.3	(17.6–25.3)
200–499	342	118	34.5	(29.5–39.8)	70	20.5	(16.3–25.1)
<200	62	18	29.0	(18.2–41.9)	12	19.4	(10.4–31.4)
ART status							
Not on ART	230	71	30.9	(25.0–37.3)	54	23.5	(18.2–29.5)
On ART	661	181	27.4	(24.0–31.0)	133	20.1	(17.1–23.4)
Duration on ART (years)							
No	230	71	30.9	(25.0–37.3)	54	23.5	(18.2–29.5)
<1	104	31	29.8	(21.2–39.6)	23	22.1	(14.6–31.3)
1–2	113	36	31.9	(23.4–41.3)	20	17.7	(11.2–26.0)
2–3	113	31	27.4	(19.5–36.6)	22	19.5	(12.6–28.0)
3–4	97	24	24.7	(16.5–34.5)	21	21.6	(13.9–31.2)
4–5	60	12	20.0	(10.8–32.3)	12	20.0	(10.8–32.3)
5+	148	43	29.1	(21.9–37.1)	30	20.3	(14.1–27.7)
Absolute CD4 cell count at start of ART (cells/mm^3^)							
200+	207	53	25.6	(19.8–32.1)	43	20.8	(15.5–26.9)
<200	363	104	28.7	(24.1–33.6)	76	20.9	(16.9–25.5)
History of opportunistic infections							
No	561	162	28.9	(25.2–32.8)	102	18.2	(15.1–21.6)
Yes	351	96	27.4	(22.8–32.3)	89	25.4	(20.9–30.2)
WHO clinical staging							
I	580	167	28.8	(25.1–32.7)	106	18.3	(15.2–21.7)
II–IV	331	90	27.2	(22.5–32.3)	85	25.7	(21.1–30.7)
Signs of RTI/STI							
No	803	233	29.0	(25.9–32.3)	165	20.5	(17.8–23.5)
Yes	109	25	22.9	(15.4–32.0)	26	23.9	(16.2–33.0)
Baseline Hybrid Capture II report							
Negative	667	182	27.3	(23.9–30.8)	114	17.1	(14.3–20.2)
Positive	245	76	31.0	(25.3–37.2)	77	31.4	(25.7–37.6)
Baseline final diagnosis							
Normal	811	225	27.7	(24.7–31.0)	160	19.7	(17.0–22.6)
CIN 1	35	9	25.7	(12.5–43.3)	10	28.6	(14.6–46.3)
CIN 2	32	13	40.6	(23.7–59.4)	8	25.0	(11.5–43.4)
CIN 3 or worse	34	11	32.4	(17.4–50.5)	13	38.2	(22.2–56.4)

ART: antiretroviral therapy; BV: bacterial vaginosis; CD4: cluster of differentiation 4; CI: confidence interval; CIN: cervical intraepithelial neoplasia; HIV: human immunodeficiency virus; HPV: human papilloma virus; RTI: reproductive tract infection; STI: sexually transmitted infection; WHO: World Health Organization.

The study participants hailed from different regions of the state of Maharashtra in India and BV was diagnosed in 191/912 (20.9%, 95% CI 18.3, 23.7) women, of them 22.0, 7.3, 21.0 and 20.0% women were from Western Maharashtra/Desh, Marathwada, Khandesh/North Maharashtra and Konkan regions, respectively. Among those diagnosed with BV, 22.9% (95% CI 18.5, 28.8), 19.6 (95% CI 14.1, 26.0) and 19.9% (95% CI 16.1, 24.2) women were from rural, semi-urban and urban settings. BV was diagnosed among 22.9% (95% CI 16.6, 30.3), 20.3% (95% CI 17.4, 23.6) and 22.4% (95% CI 13.1, 34.2) women aged <30, 30–44 and >45+, respectively. BV was diagnosed among 25.2% (95% CI 17.5, 34.4) women who had some education and 20.3% (95% CI 17.6, 23.3) women who were illiterate. BV was diagnosed among 17.1% (95% CI 14.3, 20.2) women who had a negative HC2 test report and 31.4% (95% CI 25.7, 37.6) women who had a positive HC2 test report. Among the women diagnosed with CIN 1, CIN 2 and CIN 3 or worse disease, BV was diagnosed among 28.6% (95% 14.6, 46.3), 25.0% (95% CI 11.5, 43.4) and 38.2% (95% CI 22.2, 56.4) women, respectively.

Intermediate BV was diagnosed in 258/912 (28.3%, 95% CI 25.4, 31.3) women and 27.2, 30.9, 31.9 and 34.3% women with intermediate BV were from Western Maharashtra/Desh, Marathwada, Khandesh/North Maharashtra and Konkan regions, respectively. Among the women from rural, semi-urban and urban settings, 32.5, 27.7 and 25.0% were diagnosed with intermediate BV. The distribution of intermediate BV among women with age categories was as follows: 30.6% (95% CI 23.5, 38.4), 27.3% (95% CI 24.0, 30.8) and 32.8% (95% CI 21.8, 45.4) participants with intermediate BV were <30, 30–44, 45+ years of age, respectively. Majority of the women (801/912, 87.8%) were illiterate and intermediate BV was diagnosed among 28.8% (95% CI 20.6, 38.2) women who had some education and 28.2% (95% CI 25.1, 31.5) women who were illiterate. Intermediate BV was diagnosed in 27.3% (95% CI 23.9, 30.8) women who had a negative HC2 test report and 31.0% (95% CI 25.3, 37.2) women who had a positive HC2 test report. Among the women diagnosed with CIN 1, CIN 2 and CIN 3 or worse disease, intermediate BV was diagnosed among 25.7% (95% CI 12.5, 43.3), 40.6% (95% CI 23.7, 59.4) and 32.4% (95% CI 17.4, 50.5) women, respectively.

Other participant characteristics described in Table 1 are marital status, age at first sexual contact, total number of lifetime partners, use of tobacco, total number of pregnancies, miscarriages, date of last menstruation and their HIV-related history, such as time since the diagnosis of HIV infection, baseline absolute CD4 cell count, whether on antiretroviral therapy (ART) or not on ART, absolute CD4 cell count at the start of ART, history of any opportunistic infections, their clinical staging of HIV according to the WHO and signs of RTI.

Table 2 presents the determinants of intermediate BV and BV in a univariate multinomial logistic regression analysis. Women from Marathwada region had significantly lower risk of BV (Crude OR 0.3, 95% CI 0.1, 0.8, p-value 0.015) when compared with Western Maharashtra/Desh. Women who had their first sexual intercourse after the age of 18 had significantly lower risk of BV (Crude OR 0.4, 95% CI 0.3, 0.6, p-value <0.001) as compared to those who had their first sexual intercourse before the age of 18. Women who reported use of tobacco had 1.6 times increased risk of BV (95% CI 1.0, 2.5, p-value 0.037) as compared to those who did not report use of tobacco. Women who reported more than two pregnancies had 1.8 times increased risk of BV (95% CI 1.3, 2.7, p-value <0.001). Women who were menopausal had significantly increased risk of BV (Crude OR 3.1, 95% CI 1.7, 5.7, p-value <0.001). Women who had history of opportunistic infections in the past had 1.6 times increased risk of BV (95% CI 1.1, 2.2, p-value 0.011) as compared to those who did not have it in the past. Women who were in the category of WHO clinical staging II–IV had 1.6 times increased risk of BV (95% CI 1.1, 2.2, p-value 0.009). Women who had HPV infection had 2.7 times increased risk of BV (95% CI 1.9, 3.9, p-value <0.001). Women with the diagnosis of CIN and worse disease had 3.5 times increased risk of BV (95% CI 1.5, 8.1, p-value 0.004).

**Table 2.** Determinants of bacterial vaginosis in a univariate multinomial logistic regression analysis.

Patient characteristics	Intermediate BV endpoint			BV endpoint		
	Crude odds ratio (95% CI)		p-value	Crude odds ratio (95% CI)		p-value
Geographic location in Maharashtra						
Western Maharashtra/Desh	1.0			1.0		
Marathwada	0.9	(0.5–1.7)	0.827	0.3	(0.1–0.8)	0.015
Khandesh/North Maharashtra	1.3	(0.8–2.0)	0.298	1.0	(0.6–1.7)	0.914
Konkan	1.4	(0.6–3.0)	0.389	1.0	(0.4–2.5)	0.987
Setting						
Rural	1.0			1.0
Semi-urban	0.7	(0.5–1.1)	0.126	0.7	(0.5–1.2)	0.178
Urban	0.6	(0.4–0.9)	0.007	0.7	(0.5–1.0)	0.071
Age (years)						
<30	1.0			1.0
30–44	0.8	(0.5–1.2)	0.264	0.8	(0.5–1.2)	0.295
45+	1.1	(0.6–2.2)	0.746	1.0	(0.5–2.1)	0.971
Education						
Some education	1.0			1.0
Illiterate	0.9	(0.5–1.4)	0.576	0.7	(0.4–1.2)	0.195
Marital status						
Married	1.0			1.0
Unmarried/widowed/separated	1.0	(0.8–1.4)	0.796	1.4	(1.0–1.9)	0.057
Age at first sexual intercourse						
<18	1.0			1.0
18+	0.7	(0.5–0.9)	0.015	0.4	(0.3–0.6)	<0.001
Total no. of lifetime sexual partners						
1	1.0			1.0
2+	1.1	(0.6–2.0)	0.790	1.0	(0.5–2.1)	0.912
Use of tobacco						
No	1.0			1.0
Yes	1.4	(0.9–2.1)	0.135	1.6	(1.0–2.5)	0.037
Total number of pregnancies						
0–1	1.0			1.0
2+	1.5	(1.1–2.0)	0.023	1.8	(1.3–2.7)	0.001
Miscarriages						
No	1.0			1.0
Yes	0.9	(0.6–1.2)	0.399	1.1	(0.8–1.5)	0.747
Date of last menstruation						
<12 months ago	1.0			1.0
>12 months ago	1.4	(0.7–2.7)	0.327	3.1	(1.7–5.7)	<0.001
Time since diagnosis of HIV infection (years)						
1–2	1.0			1.0
3–4	0.9	(0.6–1.4)	0.576	1.0	(0.6–1.6)	0.953
5–10	0.6	(0.4–0.9)	0.010	0.7	(0.4–1.1)	0.098
11–16	0.9	(0.5–1.5)	0.603	0.6	(0.3–1.2)	0.143
Baseline absolute CD4 cell count (cells/mm^3^)						
500+	1.0			1.0
200–499	1.7	(1.3–2.4)	0.001	1.2	(0.8–1.7)	0.404
<200	1.3	(0.7–2.4)	0.439	1.0	(0.5–1.9)	0.919
ART status						
Not on ART	1.0			1.0
On ART	0.8	(0.5–1.1)	0.147	0.7	(0.5–1.1)	0.134
Duration on ART (years)						
No ART	1.0			1.0
<1	0.9	(0.5–1.6)	0.753	0.9	(0.5–1.6)	0.712
1–2	0.9	(0.6–1.6)	0.795	0.7	(0.4–1.3)	0.216
2–3	0.8	(0.5–1.3)	0.318	0.7	(0.4–1.3)	0.260
3–4	0.7	(0.4–1.2)	0.189	0.8	(0.4–1.4)	0.433
4–5	0.5	(0.2–1.0)	0.054	0.6	(0.3–1.3)	0.245
5+	0.8	(0.5–1.4)	0.501	0.8	(0.5–1.3)	0.358
Absolute CD4 cell count at start of ART (cells/mm^3^)						
200+	1.0			1.0
<200	1.2	(0.8–1.8)	0.401	1.1	(0.7–1.7)	0.758
History of opportunistic infections						
No	1.0			1.0
Yes	1.1	(0.8–1.5)	0.717	1.6	(1.1–2.2)	0.011
WHO clinical staging						
I	1.0			1.0
II–IV	1.1	(0.8–1.5)	0.719	1.6	(1.1–2.2)	0.009
Signs of RTI/STI						
No	1.0			1.0
Yes	0.7	(0.5–1.2)	0.254	1.1	(0.7–1.8)	0.706
Baseline Hybrid Capture II report						
Negative	1.0			1.0
Positive	1.7	(1.2–2.4)	0.004	2.7	(1.9–3.9)	<0.001
Baseline final diagnosis						
Normal	1.0			1.0
CIN 1	1.1	(0.5–2.4)	0.882	1.7	(0.7–3.7)	0.218
CIN 2	2.2	(1.0–5.1)	0.054	1.9	(0.8–4.9)	0.163
CIN 3 or worse	2.1	(0.9–5.0)	0.099	3.5	(1.5–8.1)	0.004

ART: antiretroviral therapy; BV: bacterial vaginosis; CD4: cluster of differentiation 4; CI: confidence interval; CIN: cervical intraepithelial neoplasia; HIV: human immunodeficiency virus; HPV: human papilloma virus; RTI: reproductive tract infection; STI: sexually transmitted infection; WHO: World Health Organization.

When the risk of intermediate BV in the univariate multinomial logistic regression analysis was assessed, women from the urban region had significantly lower risk of intermediate BV (Crude OR 0.6, 95% CI 0.4, 0.9, p-value 0.007). Women who had their first sexual intercourse after the age of 18 had significantly lower risk of intermediate BV (Crude OR 0.7, 95% CI 0.5, 0.9, p-value 0.015). Women who reported more than two pregnancies had 1.5 times increased risk of intermediate BV (95% CI 1.1, 2.0, p-value 0.023). Women who had been diagnosed with the HIV infection in the previous 5–10 years had lower risk of intermediate BV (Crude OR 0.6, 95% CI 0.4, 0.9, p-value 0.010). Women with baseline absolute CD4 cell count between 200 and 499 had 1.7 times increased risk of intermediate BV (95% CI 1.3, 2.4, p-value 0.001) as compared to women who had their CD4 cell count more than 500. When we considered the HPV infection status and risk of intermediate BV in the univariate analysis, women who were HPV positive by HC2 test had 1.7 times increased risk of intermediate BV (95% CI 1.2, 2.4, p-value 0.004).

Table 3 presents the determinants of BV and intermediate BV in multivariate multinomial logistic regression analysis. Women who had their first sexual intercourse after the age of 18 had significantly lower risk of BV (AOR 0.4, 95% CI 0.3, 0.7, p-value <0.001) as compared to those who had their age at first sex below 18 years of age. Women with more than two pregnancies had 1.6 times increased risk of BV (95% CI 1.0, 2.5, p-value 0.038), and women who had their last menstruation more than 12 months ago had 6.2 times increased risk of BV (95% CI 2.4, 15.6, p-value <0.001). Women who tested positive by HC2 test had 2.3 times increased risk of BV (95% CI 1.4, 3.9, p-value 0.001).

**Table 3.** Determinants of bacterial vaginosis in a multivariate multinomial logistic regression analysis.

Patient characteristics	Intermediate BV endpoint			BV endpoint		
	Adjusted odds ratio (95% CI)		p-value	Adjusted^a^ odds ratio (95% CI)		p-value
Geographic location in Maharashtra						
Western Maharashtra/Desh	1.0			1.0		
Marathwada	1.1	(0.5–2.3)	0.812	0.4	(0.1–1.4)	0.158
Khandesh/North Maharashtra	1.2	(0.7–2.0)	0.483	1.0	(0.6–1.9)	0.909
Konkan	1.5	(0.6–3.6)	0.372	1.1	(0.4–3.3)	0.799
Setting						
Rural	1.0			1.0
Semi-urban	1.0	(0.6–1.6)	0.985	1.1	(0.6–2.0)	0.680
Urban	0.9	(0.6–1.4)	0.629	1.0	(0.6–1.6)	0.911
Age (years)						
<30	1.0			1.0
30–44	0.8	(0.5–1.2)	0.264	0.9	(0.5–1.5)	0.666
45+	0.6	(0.2–1.6)	0.285	0.4	(0.1–1.1)	0.084
Education						
Some education	1.0			1.0
Illiterate	1.2	(0.7–2.1)	0.567	1.1	(0.6–2.1)	0.722
Marital status						
Married	1.0			1.0
Unmarried/widowed/separated	0.8	(0.6–1.2)	0.346	0.9	(0.6–1.4)	0.721
Age at first sexual intercourse						
<18	1.0			1.0
18+	0.8	(0.5–1.2)	0.231	0.4	(0.3–0.7)	<0.001
Use of tobacco						
No	1.0			1.0
Yes	1.2	(0.7–1.9)	0.576	1.2	(0.7–2.1)	0.523
Total number of pregnancies						
0–1	1.0			1.0
2+	1.5	(1.0–2.3)	0.030	1.6	(1.0–2.5)	0.038
Date of last menstruation						
<12 months ago	1.0			1.0
>12 months ago	2.0	(0.8–5.2)	0.135	6.2	(2.4–15.6)	<0.001
Time since diagnosis of HIV infection (in years)						
1–2	1.0			1.0
3–4	1.3	(0.8–2.3)	0.295	1.3	(0.7–2.5)	0.412
5–10	0.8	(0.5–1.3)	0.406	0.9	(0.5–1.6)	0.671
11–16	1.6	(0.8–3.2)	0.165	1.0	(0.5–2.3)	0.920
Baseline absolute CD4 cell count (cells/mm^3^)						
500+	1.0			1.0
200–499	1.5	(1.0–2.2)	0.032	1.0	(0.6–1.5)	0.922
<200	0.8	(0.3–1.6)	0.470	0.4	(0.2–1.1)	0.074
Duration on ART (in years)						
No ART	1.0			1.0
<1	0.9	(0.5–1.8)	0.799	1.0	(0.5–2.0)	0.913
1–2	1.0	(0.5–1.7)	0.918	0.6	(0.3–1.3)	0.186
2–3	0.8	(0.4–1.4)	0.339	0.6	(0.3–1.2)	0.133
3–4	0.7	(0.3–1.3)	0.225	0.8	(0.4–1.5)	0.435
4–5	0.6	(0.3–1.4)	0.273	0.7	(0.3–1.7)	0.406
5+						
History of opportunistic infections						
No	1.0			1.0
Yes	1.1	(0.4–3.3)	0.872	1.7	(0.5–5.8)	0.367
WHO clinical staging						
I	1.0			1.0
II–IV	1.1	(0.4–3.5)	0.841	1.1	(0.3–3.6)	0.918
Baseline Hybrid Capture II report						
Negative	1.0			1.0
Positive	1.3	(0.8–2.1)	0.252	2.3	(1.4–3.9)	0.001
Baseline final diagnosis						
Normal	1.0			1.0
CIN 1	1.1	(0.5–2.8)	0.791	1.5	(0.6–3.9)	0.373
CIN 2	1.9	(0.7–4.7)	0.196	1.2	(0.4–3.8)	0.759
CIN 3 or worse	2.0	(0.7–5.4)	0.189	2.4	(0.9–6.5)	0.090

ART: antiretroviral therapy; BV: bacterial vaginosis; CD4: cluster of differentiation 4; CI: confidence interval; CIN: cervical intraepithelial neoplasia; HIV: human immunodeficiency virus; HPV: human papilloma virus; WHO: World Health Organization.

^a^Only factors that were significant in the univariate regression analysis were included in the multivariate model.

^b^Separate multivariate models were done for HPV genotyping results, high-risk HPV types and HPV infection.

We did not find statistically significant increased risk of intermediate BV among women with any of the characteristics except for those women who had more than two pregnancies (AOR 1.5, 95% CI 1.0, 2.3, p-value 0.030) and women who had their absolute CD4 cell count between 200 and 499 (AOR 1.5, 95% CI 1.0, 2.2, p-value 0.032).

Conversely, we also analysed data to see if women with BV had an increased risk of HPV infection (data not shown). Of the 912 women assessed, 77/191 (40.3%) women with BV were infected with high-risk HPV as assessed by the HC2 test. Women with BV had 2.7 times (95% CI 1.9–3.9, p-value <0.0001) increased risk of HPV infection in the crude multinomial multivariate analysis and 3.1 (95% CI 2.1–4.6, p-value <0.0001) times increased risk of HPV infection in the adjusted multinomial multivariate analysis (data not shown).

## Discussion

In our study, about a fifth of the HIV-infected women were diagnosed with BV. When the ‘clinical iceberg’ concept of adverse health outcomes is applied to BV, the burden of BV is likely to be much more with molecular methods than what is diagnosed by Amsel’s or Nugent’s criteria.^29^ Since BV increases the risk of other STIs, it is important that at least symptomatic women are treated appropriately following syndromic management guidelines^28^ in order to reduce their risk of acquiring other STIs and associated co-morbidities.

Factors found independently associated with BV and that increased the risk of BV in HIV-infected women were more than two pregnancies, menopause and HPV infection. Women who reported their age at first sex after the age of 18 had significantly lower risk of BV as compared to those who had first sex prior to 18 years of age. Younger age at sexual intercourse has been previously shown as a risk factor for BV.^30^ The risk of BV was significantly higher in menopausal women suggesting that reproductive hormones might be playing a protective role against BV. There have been previous reports of early menopause in HIV-infected women due to HIV-related immunodeficiency.^3^ Increasing prevalence of BV as age advances has also been reported previously among general population.^31^

Another important finding of our study is about the intermediate BV being present in about one-fourth of the women. It is estimated that about one-third of women with intermediate BV are likely to proceed to BV,^32^ and many authors feel that an intermediate BV should also be included as abnormal given the high rate of transition to BV. Although our univariate analysis showed increased risk of intermediate BV among several characteristics of HIV-infected women, the multivariate analysis showed increased risk of intermediate BV only among women who had their CD4 cell count between 200 and 499.

The prevalence of BV varies among reproductive aged women in different parts of the world but it is the most common infection among women. In a study from Kenya that evaluated 1063 HIV-infected women from 39 large HIV care programmes, 17.4% women were detected with BV^33^ whereas a study from the US has reported much higher prevalence of BV (47.8%) among HIV-infected women.^34^ A long-term follow-up study that evaluated the impact of HIV infection on BV has shown that HIV infection does not predispose to BV and BV is associated with behavioural and cultural factors.^35^

About one-third of our study participants were diagnosed with HPV infection^27^ and women with HPV infection had 2.3 times increased risk of having BV. A previous meta-analysis has confirmed that women with BV have an increased risk of HPV infection (OR 1.43; 95% CI 1.11–1.84)^36^ but this meta-analysis included only one study that was done among HIV-infected women. Because of the cross-sectional nature of our study, we do not know whether women were infected with HPV first or developed BV first. We have previously reported that women with no abortions and women with diagnosis of HIV infection in the past five years had significantly high risk of having multiple HPV infections. We have not found any common factors associated with determinants of BV and determinants of HPV infection.^37^ Previous studies and meta-analysis have reported a positive association between BV and CIN.^38^ Although we observed increased risk of BV among women with CIN 3 or worse diagnosis in the univariate analysis, it was not significant in the multivariate analysis. Previous meta-analysis has shown a positive association between BV and CIN (OR 1.51 (95% CI 1.24–1.83)).^38^

Our study has lack of availability of data on recent sexual behaviour. A previous systematic review and meta-analysis has reported that BV is transmitted to a woman by sexual contact with either a man or woman.^4^ Another limitation of our study is availability of Nugent’s scoring reports for 912/1153 participants enrolled in the main study as described previously^27^ because the Gram stain slides of the first 232/1153 (20.12%) participants enrolled in the beginning of the study could not be found. We have however confirmed that there are no differences in the characteristics of the women who had and who did not have a slide for Gram stain and subsequent Nugent’s scoring.

The prevalence of STIs is more in HIV-infected women than women who are not infected with HIV because of the same risk factors, the same route of transmission and impaired immunity among HIV-infected women.^39^ Persistence of HPV infection is necessary for the development of cervical cancer.^40^ BV can increase the risk of acquisition or reactivation of HPV infection^41^ and is also reported to be conducive to the persistence of HPV infection.^42^

To conclude, we observed high prevalence of BV as well as intermediate BV among HIV-infected women. Since BV increases the risk of acquiring STIs, persistence of HPV infection and increased risk of gynaecological outcomes, BV control among HIV-infected women should not be neglected.
